# MHC Phosphopeptides: Promising Targets for Immunotherapy of Cancer and Other Chronic Diseases

**DOI:** 10.1016/j.mcpro.2021.100112

**Published:** 2021-06-12

**Authors:** Keira E. Mahoney, Jeffrey Shabanowitz, Donald F. Hunt

**Affiliations:** 1 Department of Chemistry, University of Virginia, Charlottesville, Virginia, USA; 2 Department of Pathology, University of Virginia, Charlottesville, Virginia, USA

**Keywords:** PP2A, hepatitis HBV, hepatitis HCV, papillomavirus HPV, coronavirus, AD, Alzheimer’s disease, CagA, cytotoxin-associated antigen A, CIP2A, cancerous inhibitor of phosphatase 2A, E2F, E2 family transcription factor, EBV, Epstein Barr virus, HBV, hepatitis B virus, HCC, hepatocellular carcinoma, HCV, hepatitis C virus, HPV, human papillomavirus, MHC, major histocompatibility complex, p53, protein 53, PP1, protein phosphatase 1, PP2A, protein phosphatase 2A, pRb, retinoblastoma protein, SARS-CoV, severe acute respiratory syndrome associated coronavirus

## Abstract

Major histocompatibility complex–associated peptides have been considered as potential immunotherapeutic targets for many years. MHC class I phosphopeptides result from dysregulated cell signaling pathways that are common across cancers and both viral and bacterial infections. These antigens are recognized by central memory T cells from healthy donors, indicating that they are considered antigenic by the immune system and that they are presented across different individuals and diseases. Based on these responses and the similar dysregulation, phosphorylated antigens are promising candidates for prevention or treatment of different cancers as well as a number of other chronic diseases.

## Highlights

• Inhibition of PP2A should increase presentation of phosphorylated MHC peptides.
• Dysregulation of p53 or pRb has been shown to cause inhibition of PP2A.
• Many diseases inhibit PP2A and should present similar phosphorylated MHC peptides.
• Disease and cancer-associated PTMs are promising targets for immunotherapeutics.

## Graphical Abstract

**Figure:**
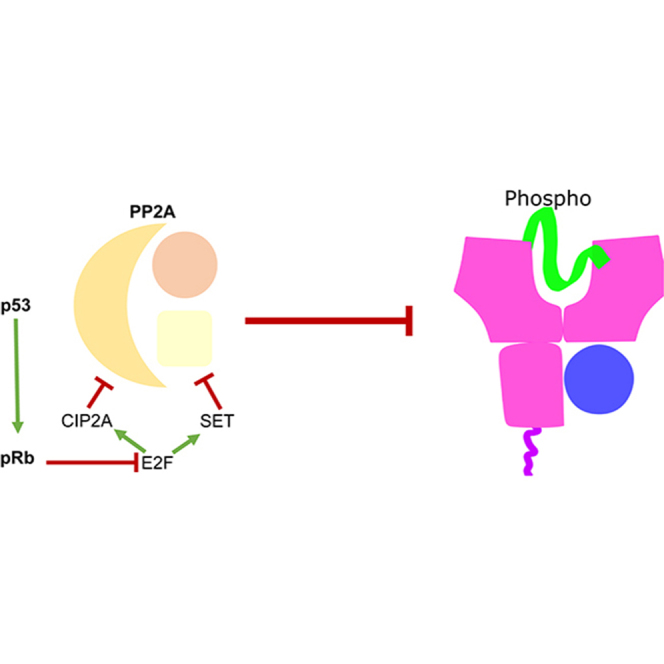

## In Brief

Although mutated antigens are currently the main focus of immunopeptidomics, disease-associated PTMs create potential targets for immunotherapeutic treatment of cancers and other diseases. We have found that many phosphorylated antigens are expressed across multiple cancers and patients. We propose that the most likely cause of this increased MHC phosphorylation is inhibition of PP2A, which is ubiquitously inhibited across cancers. If valid, a multitude of other diseases that inhibit PP2A could be targeted using the same antigen targets.

Nearly 30 years have passed since mass spectrometry was first used to understand the major histocompatibility complex (MHC) class I presentation process. After almost a year of trial and error to generate useful samples, the first MHC-associated peptide sequences were finally characterized by tandem mass spectrometry in 1992 (1). Since then, both instrument sensitivity and sample preparation techniques have improved significantly, allowing for identification of far more candidate peptides while decreasing analysis and validation times. Despite these advances, the promise of using peptides presented by the body’s own immune system as a widespread immunotherapy has encountered many challenges.

Mutated antigens have emerged at the forefront of these efforts (2, 3, 4, 5). However, finding previously undiscovered mutations is difficult and generally requires prior knowledge from RNA sequencing (4, 5, 6). The difference in MHC expression between healthy and diseased cells is small, and neoantigens are expressed at much lower levels than canonical peptides (3, 4, 5, 7). Mutated and posttranslationally modified antigens were introduced concurrently and have similar reactivity (8), whereas modified antigens are comparatively understudied (9, 10). However, disease-associated modifications are caused by dysregulated signaling pathways that are common across different individuals, cancers, and even other diseases (10, 11, 12). Many cancer treatments already use these increased modification levels, particularly phosphorylation, for targeting cancerous cells over healthy ones, but immunotherapeutic development is largely focused on mutated antigens (10, 13, 14, 15). With these increases in cellular phosphorylation we see a similar increase in presentation of phosphorylated MHC peptides. Targeting these modified peptides should allow for development of immunotherapeutics that apply to an entire HLA type instead of requiring personalized treatments.

The first phosphorylated MHC I peptide identified by mass spectrometry was published in 1998 (8). That peptide (RVAsPTSGV) has since been found in 22 samples across nine types of cancer and two HLA types (A∗02:01 and A∗68:02) (Hunt Lab Phosphopeptide List (2020), unpublished data). An elongated version, RVAsPTSGVK, has been found in 16 samples, 4 cancers, and two HLA types (A∗03:01 and A∗11:01) (Hunt Lab Phosphopeptide List (2020), unpublished data). Since 1998, our laboratory has identified more than 2500 potential targets across 15 types of cancer; approximately 1000 of these have been found in multiple cancer types (Hunt Lab Phosphopeptide List (2020), unpublished data). (16) Some phosphopeptides have been found in up to 13 types of cancer and ~40 samples (Hunt Lab Phosphopeptide List (2020), unpublished data). Our current methodology for phosphopeptide enrichment and analysis can produce anywhere from tens to hundreds of MHC I phosphopeptide identifications using 100 to 500 mg of cancerous tissue (17, 18). Studies looking for mutated antigens use similar amounts of tissue and generally identify under 10 mutated antigens, most of which are specific to the tissue and patient (7, 19). Conversely, more than 50% of the phosphopeptide identifications for any given sample are generally already identified in another sample (depending on the prevalence of the HLA types) (Hunt Lab Phosphopeptide List (2020), unpublished data). Approximately 80% of phosphopeptides tested can generate central memory T cell responses from healthy donors (Hunt Lab Phosphopeptide List (2020), unpublished data). This response indicates that their immune system considers these peptides to be antigenic and that the response has overcome central tolerance (20, 21). Therefore, peptides that generate a healthy donor response are promising targets for immunotherapy of multiple cancers, as well as any other disease that causes their expression (20, 21).

Research indicates that many class I MHC phosphopeptides are uniquely expressed on diseased cells and are potential targets for the immunotherapy of hepatocellular cancer, breast cancer (22), melanoma (23), colorectal cancer (16), leukemias (21), and other cancers (24, 25, 26, 27). Of particular note are results obtained in a recent preclinical trial that used two of our class I MHC phosphopeptides to treat high-risk melanomas (28). T cell responses to one peptide were observed in 5 of 12 patients. Another peptide generated T cell responses in 2 of 12 patients. Adverse effects were minimal. Two additional clinical trials that use multiple class I MHC phosphopeptides to treat acute myeloid leukemia and colorectal cancer are in the planning stages.

Two important questions are posed by these findings: (a) why do dysregulated cell signaling pathways generate the same phosphopeptide antigens on multiple types of cancer and (b) why do healthy blood donors, with no sign of autoimmune disease, have central memory T cells that recognize and kill cells that present these same class I MHC phosphopeptide antigens? Answers to both questions likely involve three major tumor suppressor proteins: protein phosphatase 2A (PP2A) (29, 30, 31, 32, 33, 34, 35, 36), retinoblastoma protein (pRb) (37), and tumor suppressor protein 53 (p53) (38).

## Oncogenic Proteins and Phosphorylation

Phosphopeptide antigens are derived from dysregulated, cell signaling pathways common to many different cancers. In normal cells, phosphorylation is generally a brief event. However, dysregulation in cancerous cells extends the length of phosphorylation, allowing the proteasome sufficient time to degrade the phosphorylated protein forms and present phosphopeptide antigens on class I MHC molecules. Since the same cell signaling pathways are also dysregulated during many viral and bacterial infections, many of the same phosphopeptide antigens should be presented. Therefore, these antigens could be employed for immunotherapy of, or vaccination against, both infectious agents and cancer.

PP2A is the most abundant serine/threonine phosphatase in the cell (comprising ~0.1%–1% of all cellular proteins) and plays a major role in the regulation of many important cell signaling pathways (*e.g.*, Wnt, P13K/Akt, MAPK, and c-Myc) that control cell proliferation, transformation, and apoptosis (29, 30, 31, 32, 33, 34, 35, 36). PP2A exists as a heterotrimeric complex consisting of a scaffolding subunit (A), regulatory subunit (B), and a catalytic subunit (C). There are two isoforms for subunit A, two isoforms for subunit C, and four classes of subunit B, each of which has 2 to 5 isoforms and additional splice variants. The regulatory subunits allow for cellular localization and impart specificity toward different substrates (29). As the most prolific and abundant phosphatase, altering the activity of PP2A should have the greatest effect on phosphorylation states (31, 32).

A similar phosphatase, protein phosphatase 1 (PP1), is the next most prolific phosphatase. PP1 lacks a scaffolding subunit but has four isoforms of the catalytic subunit and about 60 known regulatory subunits (39, 40). The catalytic subunits of the two phosphatases have a high degree of sequence homology but have different substrate binding motifs (41). Both are also reversibly phosphorylated near the C terminus to control activation (42). Since the catalytic subunits are so similar, inhibitors that target the PP2A catalytic subunit or phosphate frequently have a significant effect on PP1 as well (34, 43).

PP1 and PP2A together are responsible for the vast majority of dephosphorylation in the cell, so dysregulation of one or both of them would have a significant effect on cellular phosphorylation (44, 45). Many phosphorylation sites may be dephosphorylated by multiple phosphatases, but a reduction in PP2A activity would cause a large reduction in overall phosphatase activity. Therefore, even if there are redundant phosphatases for a site, a large reduction in the total available phosphatase for a site should cause an extended phosphorylation event. Although kinases are also dysregulated in cancer, each one is responsible for a far smaller subset of sites, meaning they individually have a much smaller effect on cellular phosphorylation. For example, all 15 members of the protein kinase C family, the most prolific kinase family, are together predicted to be responsible for ~20% of phosphorylation sites (46, 47). Based on their relative levels of control over phosphorylation, inhibition of PP2A and/or PP1 should increase cellular phosphorylation much more than overactivation of any kinase or kinase family.

PP2A inhibition is accepted as a prerequisite for cellular transformation and is likely responsible for many of the phosphorylated antigens that we have observed across cancers (48, 49, 50, 51). Accordingly, it has become a major area of research in the development of cancer therapeutics (13, 29, 52, 53). PP2A can be directly inactivated by somatic mutation and phosphorylation or demethylation near the C terminus of the catalytic subunit (29, 42). Somatic mutations are found in about 8% of cancer cases, with the scaffolding subunit as the most common site (20%) (29). Mutation of the scaffolding subunit generally causes an inability to bind regulatory subunits, thereby preventing the phosphatase from localizing or associating with its targets (29). Although these direct inhibitions are present in some cancers, PP2A is more commonly inactivated by upregulation of endogenous PP2A inhibitors: protein SET (also known as inhibitor 2 of PP2A) and cancerous inhibitor of phosphatase 2A (CIP2A). CIP2A, the more heavily studied of the two, is overexpressed in ~40% to 90% of patients across different cancer types (54, 55, 56, 57, 58). Both CIP2A and SET appear to inhibit PP2A activity by binding to the catalytic subunit, preventing it from associating with the rest of the holoenzyme (59, 60, 61). High CIP2A and/or SET levels have repeatedly been associated with worse prognosis across cancers (52, 54, 58, 62, 63). Overexpression of these endogenous inhibitors is generally caused by inhibition of another major tumor suppressor, pRb (31, 59).

pRb was proposed by Alfred Knudson in 1971 as a ubiquitous mutation in patients with retinoblastoma (64). It is one of the earliest discovered oncogenic proteins and its dysregulation has since been observed in multiple other cancers. pRb is a major regulator of the cell cycle and is mutated or functionally inactivated in most human cancers (37). pRb represses gene transcription required for cell growth by directly binding to the transactivation domains of the E2F family of transcription factors. pRb also represses transcription by binding to proteins that are involved in nucleosome remodeling, histone acetylation/deacetylation, and methylation. pRb becomes inactive when it is phosphorylated by cyclin-dependent kinases. Hyperphosphorylated pRb dissociates from E2 family transcription factors (E2Fs) and allows the cell to enter S-phase with high levels of transcription. Synthesis of endogenous PP2A inhibitors CIP2A and inhibitor 2 of PP2A (I2PP2A/SET) is upregulated when pRb is dissociated from E2F. Since reactivation requires dephosphorylation by PP2A, reactivation becomes very difficult with even a small amount of dysregulation.

Tumor suppressor p53, perhaps the most heavily studied oncogenic protein, is a sequence-specific DNA-binding protein that regulates transcription and promotes cell cycle arrest and apoptosis (38, 65). It can activate DNA repair proteins when DNA has sustained damage, arrest growth by holding the cell cycle at the G1/S regulation point to give the repair machinery time to fix DNA sequence errors, and initiate programmed cell death (apoptosis) if the damage proves to be irreparable. p53 is activated by phosphorylation and degraded in the proteasome when it is ubiquitinated by the ubiquitin ligase Mdm2 (66). PP2A is responsible for a dephosphorylation at Thr55 that activates p53 and for a number of phosphorylation sites on Mdm2 that allow for appropriate p53 regulation (66, 67, 68). Mutation of p53 also occurs in most human cancers (38).

PP2A is responsible for activating both pRb and p53 through dephosphorylation (36). Thus, its inhibition causes a decrease in the essential functions of pRb and p53 in maintaining cell homeostasis (65, 67). Inactivation of pRb increases transcription of CIP2A and SET, leading to PP2A inhibition. Inactivation of p53 prevents cell cycle arrest after DNA damage and lowers production of p21. The lack of p21 increases cyclin-dependent kinase 2 activity, resulting in pRb hyperphosphorylation and dissociation from E2F (69). E2F is then active and able to increase transcription of CIP2A and SET. Inactivation of p53 also allows for mutations to continue unchecked, increasing the chance of mutating further tumor suppressor proteins.

Many cell functions have redundant pathways, allowing individual proteins to malfunction without causing overall cell malfunction. However, these three proteins have limited redundancy, so interruption of their function leads to cellular dysfunction and, in many cases, oncogenesis. Furthermore, interference with one of these proteins often leads to interference with the other two, causing progressively greater changes to cellular functions that increase cell proliferation and immune evasion.

**Fig. 1:**
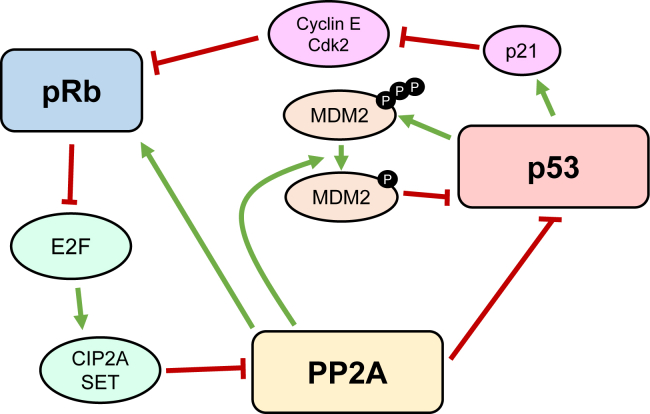
**Inhibition of any one of tumor suppressor proteins pRb, p53, or PP2A can cause inhibition of the other tumor suppressors, either directly or through dysregulation of connected pathways**.

Owing to their relationships, interference with any of these proteins should also cause reduced activity of the most prolific phosphatase (Fig. 1). Disruption of PP2A causes an extended phosphorylation event, allowing enough time for expression of phosphorylated MHC peptides. Therefore, disruption that reduces PP2A activity allows us to find cancer-specific MHC phosphopeptides expressed across multiple cancer and/or tissue types. Based on the sites it is responsible for and the ubiquity of its inhibition, PP2A (perhaps in combination with PP1) is the most likely cause of increased cellular phosphorylation as well as phosphoantigen presentation.

## Disease-associated PP2A Inhibition

**Fig. 2:**
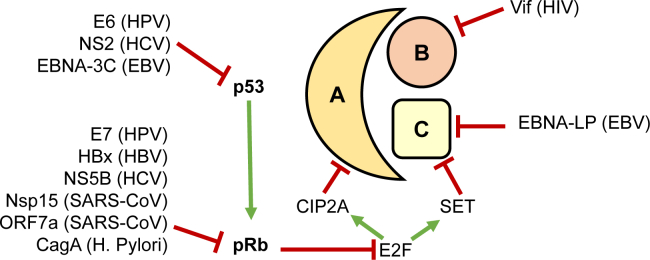
**Viral proteins that lead to inhibition of specific subunits of PP2A.** Inhibition of the scaffolding (A) or catalytic (C) subunits would affect PP2A activity much more than inhibition of the regulatory (B) subunit, which would affect only a subset of proteins.

Since p53, pRb, and PP2A are important in pathways that are responsible for the success of any disease (proliferation and avoidance of immune detection), they are also common targets for some of the most chronic and difficult-to-treat diseases, regardless of the pathogen (36, 39, 65). If this reasoning is valid, any disease that targets any combination of p53, pRb, or PP2A has the potential to produce the same MHC-associated phosphopeptides as cancerous cells (Fig. 2). In other words, an immunotherapy that utilizes MHC-associated phosphopeptides should work against diseases that targets any of these three proteins. In this section, we will discuss several diseases that target p53, pRb, and/or PP2A. Since inhibition of these proteins should lead to extended protein phosphorylation through phosphatase inhibition, it should also lead to the presentation of phosphorylated MHC I peptides.

### Hepatitis B and C Viruses

Worldwide, there are 140 million people infected with hepatitis C virus (HCV) and more than 250 million people with hepatitis B virus (HBV) (70). Both viruses can cause hepatocellular cancer. HCV consists of a single-stranded RNA (9600 nucleotide bases) surrounded by a protected shell of proteins. The viral RNA codes for a single polyprotein (~3000 aa) that is posttranslationally cleaved into two highly glycosylated structural proteins E1 and E2, a transmembrane protein p7, and six nonstructural, accessory proteins: NS2, NS3, NS4A, NS4B, NS5A, and NS5B. HCV does not integrate its genome into the host chromosomal DNA (70). It exhibits a high mutational rate and deregulates many host cellular processes. An accessory protein, NS5B, forms a complex with the retinoblastoma tumor suppressor protein (pRb) that is then targeted for degradation in the proteasome following ubiquitination by the E6-associated protein (E6AP) (71). Expression of another member of the pRb family, p130, is downregulated by the HCV core protein that triggers hypermethylation of the promoter region of the corresponding gene (72). Another accessory protein, NS2, sequesters p53 to the cytoplasm and prevents it from monitoring DNA damage and triggering cell apoptosis (73). The expected result would be high levels of gene transcription, likely including production of CIP2A, and uncontrolled cell division.

The hepatitis B virus (HBV) is a partially double-stranded DNA virus that replicates *via* reverse transcription. The two DNA chains contain ~3200 and ~2300 nucleotides, respectively. The genome contains four overlapping reading frames that code for the viral coat protein (capsid), surface proteins (envelope), reverse transcriptase, and the small (17.4 kDa) regulatory oncoprotein, HBx (41). In hepatocellular carcinoma (HCC) cases caused by HBV, the virus frequently (86.4%) integrated into the host genome (74). HBx activates the E2F1 group of transcription factors by upregulating kinases that phosphorylate and inactivate pRb (75). HBx is also known to block apoptosis of HBV-infected cells by several different mechanisms (76).

**Fig. 3:**
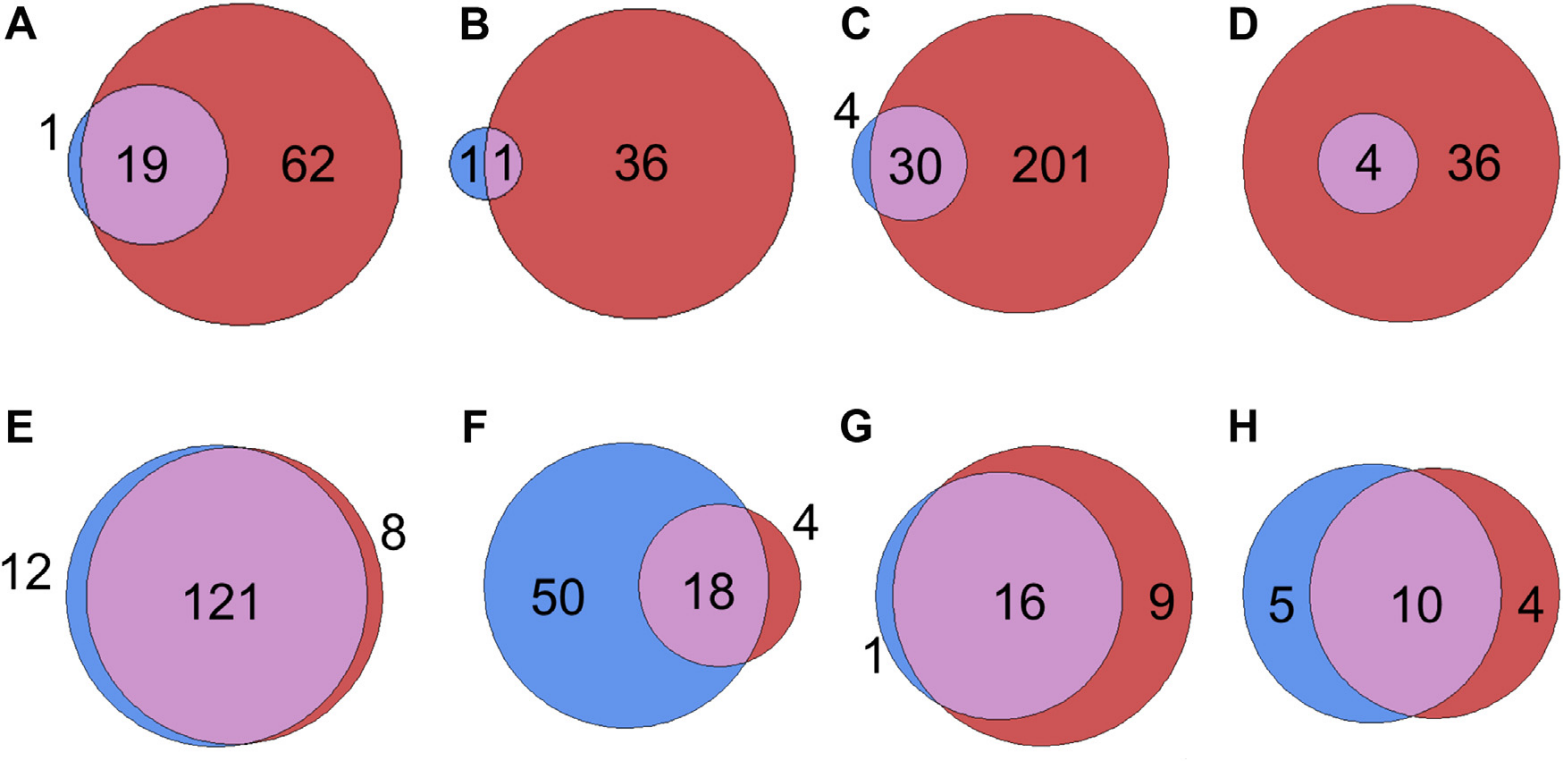
**Overlap between MHC phosphopeptide expression on tumor tissue (*red*) compared with the surrounding tissue (*blue*).** Margin tissue can express some phosphopeptides owing to cellular changes prior to transformation. However, the overlap between tumor and margin tissue is much more pronounced in tumors caused by hepatitis B (*E* and *H*) and/or hepatitis C (*F*–*H*) than it is in samples without a known cause (*A* and *B*) or adenoma transformation (*C* and *D*).

Our theory that this should lead to increased phosphorylation of MHC peptides on the surface is supported by comparisons of tumor margin tissue from patients with HCC. Margin tissue around tumors is generally compromised in some way, either because the margin and tumor were not cleanly separated or because the tissue surrounding the tumor suffers from the same conditions that caused the tumor (*e.g.*, alcoholic liver disease). In a study comparing phosphorylation between HCC cases, margin tissue from five patients without these diagnoses (caused by adenoma transformation) (Fig. 3, *C* and *D*) or unknown etiology (Fig. 3, *A* and *B*) expressed, on average, 11% (0%–25%) of the phosphorylated MHC peptides seen on the corresponding tumor tissue (N. Buettner *et al*., unpublished data). In contrast, margin tissue from five patients with HBV and/or HCV shows far more phosphorylation, ranging from 55% to 675% of the corresponding tumor’s expression (Fig. 3, *E*–*H*) (N. Buettner *et al*., unpublished data). This supports the theory that hepatitis infection causes increased phosphopeptide expression prior to transformation.

### Human Papillomavirus

Human papillomavirus (HPV) infects the basal cells of human epithelia and is the main causative agent for many human tumors including cervical, anal, and oral cancers (77, 78). There are close to 200 different HPV types, many of which are oncogenic. The oncogenic strains are also referred to as “high risk” and produce the same proteins as the low-risk variants, but with sequence variations that alter activity in a way that can lead to cellular transformation (79, 80). Most notably, HPV-16 and HPV-18 are responsible for the majority of cases of cervical cancer, the second most common cancer in women worldwide (78). Cells infected with high-risk HPV are often able to carry out transformation without cell lysis or inflammation, allowing it to avoid detection by the immune system (78).

Oncogenic variants of HPV encode a 98-residue phosphoprotein (E7) that binds to the active, unphosphorylated form of pRb as well as related proteins p130 and p107 and targets them for degradation in the proteasome (80, 81). When the E7 protein targets pRb for degradation, E2F1, a member of the E2F1-3 transcription factor family that was repressed by pRb, now becomes activated and upregulates expression of the protein CIP2A (50, 82, 83). The E7 protein produced by low-risk strains of the virus is less phosphorylated than the high-risk variants and has significantly lower affinity for pRb (79, 84). Another HPV accessory protein, E6, upregulates the DNA cytosine deaminase APOBEC-3B (A3B), an enzyme that converts cytosine to uracil and causes hypermutation of the viral DNA (80, 85, 86). Normally, this would activate the tumor suppressor protein p53, to trigger apoptosis. Unfortunately, the 158-residue E6 protein and the E6AP cellular protein form a complex that allows them to bind p53 and target it for ubiquitination and degradation in the proteasome (80). The low-risk variant of E6 is still able to associate with p53 but is unable to target it for degradation (87).

### Epstein Barr Virus

More than 90% of adults in the world have been infected with the Epstein Barr virus (EBV) (also known as human herpesvirus 4), and most continue to have a lifelong dormant infection (88). EBV infects both B cells and epithelial cells. The reservoir for the latent virus is primarily resting, central memory B cells. EBV is known to cause infectious mononucleosis and a variety of cancers such as Hodgkin’s lymphoma, Burkitt’s lymphoma, gastric cancer, and nasopharyngeal carcinomas (89, 90).

The virus is composed of a double-stranded DNA helix that codes for 85 proteins and is surrounded by a protein nucleocapsid and an envelope of both lipids and glycoproteins. Regulatory proteins of note include six nuclear antigens (EBNA-1, -2, -3A, -3B, 3C, and the EBV nuclear antigen-leader protein, EBNA-LP) plus three EBV latent membrane proteins (LMP-1, -2A, and -2B) (91). EBNA-3C (also known as EBNA-6) binds the mitochondrial ribosomal protein MRPS18-2 and targets it to the nucleus, where it binds to the pRb and liberates the E2F1 group of transcription factors (92). EBNA-3C can also recruit the SCFSkp2 ubiquitin ligase complex that mediates ubiquitination and degradation of pRb, resulting in high levels of transcription (93). EBNA-3C also enhances the intrinsic ubiquitin ligase activity of Mdm2 toward p53, which in turn facilitates p53 ubiquitination and degradation (94). As with the other viruses, this should liberate the transcription factors that upregulate expression of endogenous PP2A protein inhibitors SET and CIP2A. In addition, a truncated form of EBNA-LP has been shown to interact with and bind to the catalytic subunit of PP2A (95).

### Human Immunodeficiency Virus

The human immunodeficiency virus (HIV-1) is a retrovirus that infects CD4+ T cells, macrophages, and dendritic cells and eventually causes AIDS. More than 40 million people worldwide are infected with the virus. HIV-1 is composed of two copies of single-stranded RNA that produce 16 proteins (96). Four HIV accessory proteins, Vif, Vpr, Nef, and Vpu, share the ability to target cellular proteins for proteasomal degradation and are essential for pathogenesis *in vivo* (96, 97). Recently, the accessory protein Vif was discovered to be necessary and sufficient for culin-5 dependent ubiquitination and proteasomal degradation of all members of the B56 family of regulatory subunits for PP2A (69, 98). Inhibition of PP2A by Vif produced hyperphosphorylation of cellular proteins that mirrored previously reported changes seen when PP2A was treated with the small molecule inhibitor okadaic acid in transformed cells (98, 99).

### Coronaviruses

Severe acute respiratory syndrome associated coronaviruses (SARS-CoVs) are highly infectious single-stranded RNA viruses that cause respiratory illness. SARS-CoV-1 was the first pandemic of the 21st century, infecting approximately 8000 people from 29 countries between 2002 and 2004. A similar but far more infectious virus emerged in 2019, the novel coronavirus SARS-CoV-2. Infecting over 70 million people worldwide in 2020, SARS-CoV-2 has become a far worse pandemic. Since SARS-CoV-2 is relatively novel, there is little published research regarding its cell dysregulation and long-term effects. However, there are several papers that show PP2A or pRb dysregulation in SARS-CoV-1 infected cells, and studies linking them with SARS-CoV-2 are beginning to emerge.

SARS-CoV-1 and SARS-CoV-2 encode 28 and 26 proteins, respectively (100). Nonstructural protein 15 (Nsp15) is a 180-residue protein that has 89% sequence homology between SARS-CoV-1 and SARS-CoV-2 (100). Nsp15 from SARS-CoV-1 was previously found to target pRb for degradation through binding with a LXCXE/D motif (101). This is the same motif that the HPV E7 protein uses for targeting pRb (102, 103). This motif is conserved in SARS-CoV-2 (L331-D335 in both proteins) and should serve the same function. Another SARS-CoV-1 protein, ORF7a, was also found to inhibit pRb through hyperphosphorylation (104). In addition, the scaffolding subunit of PP2A was found to be three times less abundant in cells infected with either SARS-CoV, although the cause is undetermined (105). New research indicates that the SARS-CoV-2 spike glycoprotein contains motifs for binding to the B56α subunit, which would cause further inhibition of PP2A (106).

### Helicobacter pylori

*Helicobacter pylori* is a gastrointestinal bacterium that is present in about 50% of the population. In 2005, the Nobel prize in physiology or medicine was awarded for discovery of its link with gastritis and peptic ulcer disease. Certain strains of the bacteria are known to release the protein cytotoxin-associated antigen A (CagA). CagA-positive strains have a stronger link with development of peptic ulcers and are considered oncogenic owing to the protein’s activation of numerous pathways associated with oncogenesis, including MAPK, NF-κB, Wnt/β-catenin, and PI3K (107). CagA has been linked with hyperphosphorylation (and the resultant deactivation) of pRb, causing increased levels of E2F1 transcription factors and greater cell proliferation (108). CagA has also been shown to increase CIP2A expression, which is likely caused by the pRb inactivation (109).

### Fusobacterium nucleatum

*Fusobacterium nucleatum* is a gram-negative anaerobe that is usually the most abundant bacterium in the oral cavity and plays a key role in the development of dental plaque and a number of oral diseases (110). However, it also flourishes outside the oral cavity and is responsible for many infections. Presence of this bacteria has been strongly connected to colorectal carcinogenesis and progression (111, 112, 113, 114). It has also been linked to esophageal (115), intestinal (116), and breast (117) cancers. It is known to promote colorectal carcinogenesis by modulating E-cadherin/β-catenin signaling (111, 114). The *F. nucleatum* genome codes for the protein FadA, which binds to E-cadherin on colorectal cells and mediates attachment and invasion of the bacterium. Both FadA (114) and the *F. nucleatum* lipopolysaccharide (118) have been reported to activate β-catenin signaling, which should result in generation of CIP2A.

### Neurodegenerative Diseases

Abnormal hyperphosphorylation of protein Tau is the main hallmark of a number of neurodegenerative disorders called tauopathies. The most studied of these is Alzheimer’s disease (AD), but they include a number of other dementias and neurodegeneration following traumatic brain injury or encephalopathy (119). In these diseases, Tau is 3 to 4 times more phosphorylated than it is in healthy cells. This hyperphosphorylation leads to microtubule disorganization, protein aggregation, and cell death (120). PP2A is responsible for 70% of phosphatase activity for protein Tau and has become a major topic of study in the field (121). Inhibition of PP2A using okadaic acid has been shown to induce p-Tau formation as well as other characteristics of tauopathies including cognitive impairment, protein aggregation, and cell death (120). Increased pRb phosphorylation and decreased PP2A methylation have been observed in AD, both of which should ultimately cause PP2A inhibition (33). Increased levels of both CIP2A and SET colocalized with increased p-Tau levels in stains of AD tissue (122).

Immunopeptidomic studies of brain tissue are difficult and are not frequently attempted (123). Most obviously, the brain is not a common operation site and is unlikely to be donated. In addition, brain tissue has low MHC expression, about 70 times lower than most other tissue types (124). However, we were able to identify two phosphorylated MHC I peptides in a 400-mg cadaverous neurodegenerative sample. Both were previously found in multiple cancers and generate responses from healthy donor memory T cells. We have not identified any phosphorylated antigens on cadaverous tissues from healthy donors.

## Conclusions

From our studies over the past 20 years, we have observed that phosphorylated MHC peptides are expressed primarily on diseased tissue and across multiple cancer types. Since PP2A is prolific and its inhibition is heavily linked with cancer, it is the most likely cause of the increased phosphorylated antigen expression seen in cancer tissues. If this increase in phosphopeptide antigens is caused by PP2A dysregulation, then a treatment developed using these phosphopeptides could apply to any disease with the same dysregulation. Expression of phosphorylated MHC antigens by cells infected with these pathogens would explain why healthy donors with no prior cancer diagnosis have memory T cells that recognize these antigens. That is, common viruses such as EBV may give the immune system an opportunity to recognize these peptides as antigenic, resulting in memory T cells.

Even though the limited evidence we have managed to collect thus far is promising, far more research is needed to confirm our theories. Generating the quantities of cells needed to confirm whether other diseases cause expression of cancer-linked phosphorylated antigens is difficult. Although infection of cancerous cell lines is commonplace, efficient infection of high quantities of healthy cells is difficult. In addition, confirmation would require cells that are infected but not cancerous. Since many of these viruses are oncogenic, the line between infection and cancer may be uncertain. For example, EBV is frequently used to immortalize cells, so it is difficult to define when to consider the cells transformed rather than merely infected.

Despite these challenges, modified antigens are appealing targets for immunotherapeutic treatments for many reasons. Not only are they expressed across many different cancers and individuals, but they also have the potential to allow treatment of many other chronic and debilitating diseases. Since this is a less common approach for antigen identification, there is ample space for discovery of novel antigens or diseases. The concept could be further expanded to study other posttranslational modifications, such as methylation and glycosylation, which are also dysregulated across different cancers and diseases.

## Conflict of interest

J. S. and D. F. H. have financial interest in Agenus, Inc.
